# Scanning Electron-Assisted Dielectric Microscopy Reveals Autophagosome Formation by LC3 and ATG12 in Cultured Mammalian Cells

**DOI:** 10.3390/ijms22041834

**Published:** 2021-02-12

**Authors:** Tomoko Okada, Toshihiko Ogura

**Affiliations:** Health and Medical Research Institute, National Institute of Advanced Industrial Science and Technology (AIST), Central 6, Tsukuba, Ibaraki 305-8566, Japan; t.okada@aist.go.jp

**Keywords:** autophagy, scanning electron microscopy, dielectric microscopy, LC3, Atg12, actin, gold colloid

## Abstract

Autophagy is an intracellular self-devouring system that plays a central role in cellular recycling. The formation of functional autophagosomes depends on several autophagy-related proteins, including the microtubule-associated proteins 1A/1B light chain 3 (LC3) and the conserved autophagy-related gene 12 (Atg12). We have recently developed a novel scanning electron-assisted dielectric microscope (SE-ADM) for nanoscale observations of intact cells. Here, we used the SE-ADM system to observe LC3- and Atg12-containing autophagosomes in cells labelled in the culture medium with antibodies conjugated to colloidal gold particles. We observed that, during autophagosome formation, Atg12 localized along the actin meshwork structure, whereas LC3 formed arcuate or circular alignments. Our system also showed a difference in the distribution of LC3 and Atg12; Atg12 was broadly distributed while LC3 was more localized. The difference in the spatial distribution demonstrated by our system explains the difference in the size of fluorescent spots due to the fluorescently labelled antibodies observed using optical microscopy. The direct SE-ADM observation of cells should thus be effective in analyses of autophagosome formation.

## 1. Introduction

Autophagy is a process of the self-devouring of unnecessary and dysfunctional cellular components such as protein aggregates and damaged organelles as well as invading bacteria, viruses, and protists [1,2,3,4,5]. Such materials are incorporated into and enclosed by a double-membraned autophagosome, which later fuses with the lysosome [2,3,6]. The formation of functional autophagosomes depends on the hierarchically ordered activities of many autophagy-related proteins [7,8]. One category of these proteins includes ubiquitin-like proteins of the microtubule-associated proteins 1A/1B light chain 3 (LC3) family [9,10] and the conserved autophagy-related gene 12 (Atg12) [11,12]. When autophagy is induced, LC3 is conjugated to phosphatidyl-ethanolamine at the autophagosome-forming site [10,12]. This conjugation plays a crucial role in controlling the membrane dynamics, while Atg12 is constitutively conjugated to Atg5 [12,13,14]. As revealed by immunofluorescence microscopy and electron microscopy, the Atg12–Atg5 conjugate is necessary for LC3 lipidation and, therefore, essential for autophagosome formation [9,15,16,17,18,19]. Many studies have demonstrated that autophagosomes are constructed via cooperation between LC3 and Atg12 [15,16,17,18,19]. However, direct observations are required of cells in culture medium.

Recently, we developed a novel imaging technology, scanning electron-assisted dielectric microscopy (SE-ADM), which enables the observation of intact cells, bacteria, and protein particles in a medium with extremely low radiation damage and without requiring staining and fixation [20,21]. Our system can produce high-contrast images of untreated biological specimens under aqueous conditions [21,22,23,24,25]. The biological samples to be observed are enclosed in a liquid holder composed of two silicon nitride (SiN) films, the upper film of which is coated with a tungsten (W) layer. When an electron beam (EB) is applied to the W-coated SiN film, EB is scattered and mostly absorbed by the W layer, so the samples are protected from EB-caused damage and we can observe the same place of the sample repeatedly (see Supplementary Figure S1) [20,21,22]. In a previous study, we clearly observed the unstained actin meshwork structure in cells in aqueous media using the SE-ADM system [24].

In this report, we show that our SE-ADM system enables the observation of autophagosomes in cells labelled in the culture medium with antibodies conjugated to colloidal gold particles.

## 2. Results

### 2.1. Localisation of LC3 and Actin in Cultured Cells Using Fluorescence Microscopy

**Figure 1:**
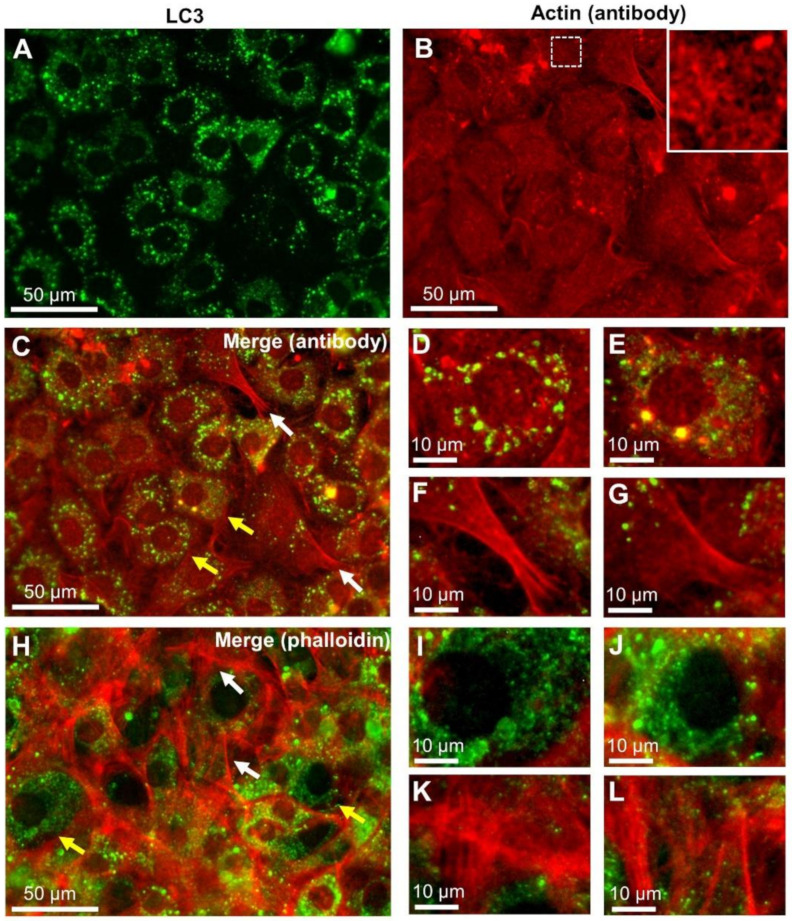
Observation of microtubule-associated proteins 1A/1B light chain 3 (LC3) and actin double-stained fluorescence images of rat embryonic fibroblasts (REF) cells. (**A**) Indirect fluorescein isothiocyanate (FITC) immunofluorescence labelling of LC3 using optical microscope (OM) with a green fluorescence filter (see Methods; 400× magnification). (**B**) Indirect rhodamine immunofluorescence labelling of actin viewed through a red fluorescence filter (see Methods; 400× magnification). An enlarged image of the white framed area is shown in the upper corner. (**C**) A merged fluorescence image of LC3 and actin. (**D**,**E**) Enlarged images of the LC3-rich areas (indicated by yellow arrows in **C**). (**F**,**G**) Enlarged images of the LC3-poor areas (indicated by white arrows in (**C**)) showing many filamentous actin bundles. (**H**) A merged image of LC3 and phalloidin-labelled filamentous actin. (**I**,**J**) Enlarged images of the LC3-rich areas (indicated by yellow arrows in (**H**)). (**K**,**L**) Enlarged images of the LC3-poor areas (indicated by white arrows in (**H**)). Scale bars: 50 μm in (**A**–**C**,**H**), 10 μm in (**D–G**,**I–L**).

**Figure 2:**
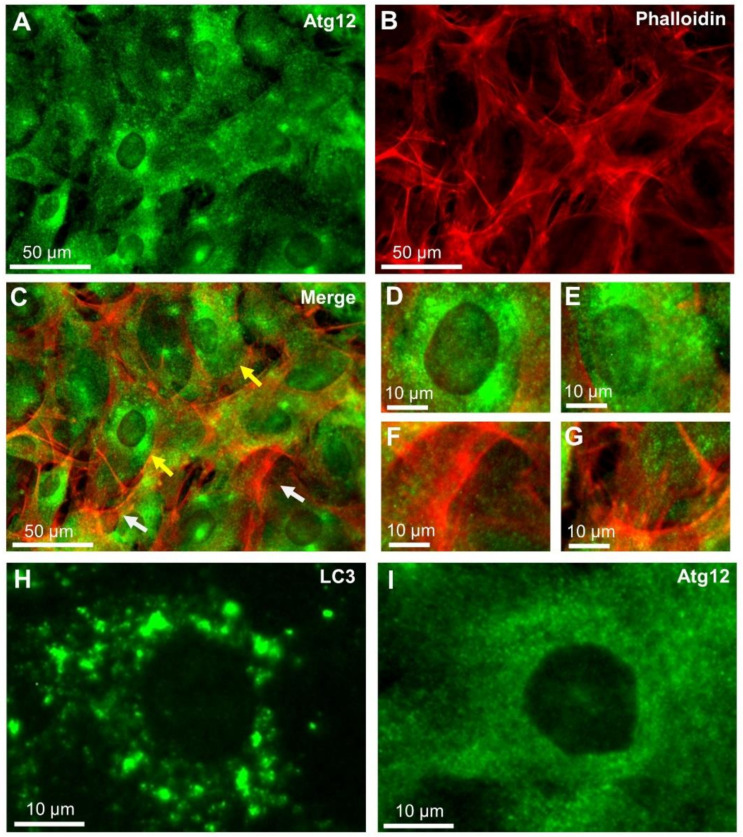
Observation of Atg12 and actin double-stained fluorescence images of REF cells. (**A**) Indirect FITC immunofluorescence labelling of Atg12 using OM with a green fluorescence filter (see Materials and Methods; 400× magnification). Small fluorescence spots near the nucleus are Atg12. (**B**) A fluorescence image of actin filament stained by rhodamine-conjugated phalloidin, viewed through a red fluorescence filter (400× magnification). (**C**) A merged fluorescence image of Atg12 and actin. (**D**,**E**) Enlarged images of the Atg12-rich areas (indicated by yellow arrows in (**C**)). (**F**,**G**) Enlarged images of the Atg12-poor areas (indicated by white arrows in (**C**)). Fluorescence spots of Atg12 are rare near the filamentous actin. (**H**,**I**) Enlarged images of the LC3 and Atg12 fluorescence spots, respectively. Scale bars: 50 μm in (**A**–**C**), 10 μm in (**D**–**I**).

To examine the localization of LC3 and actin in rat embryonic fibroblasts (REF) cells [26], we obtained fluorescence images of REF cells labelled with antibodies targeting LC3 and actin (Figure 1 and Figure 2). The nuclei of the stained cells were clearly visible using the fluorescence optical microscope (OM). In the LC3 fluorescent image (Figure 1A), spherical spots with a diameter of about 1 μm were seen to be dispersed around the nucleus. The actin fluorescence image showed meshwork structures near the nucleus and filamentous structures in the periphery (Figure 1B). When the LC3 and actin fluorescence images were merged, LC3 was observed to colocalize with the actin meshwork structure as perinuclear actin (Figure 1C). Panels D and E of Figure 1 are enlarged images of the regions indicated by yellow arrows in Figure 1C. Conversely, the actin filaments in the periphery did not colocalize with LC3. Similarly, we did not observe any colocalization of LC3 with the phalloidin-labelled actin filaments (Figure 1H–L).

### 2.2. Localisation of Atg12 and Actin in Cultured Cells Using Fluorescence Microscopy

Next, we examined the localization of Atg12 and actin by double-stained fluorescence imaging. The fluorescein isothiocyanate (FITC)-labelled Atg12 were found to be dispersed near the nucleus as small spots with diameters far below 1 μm (Figure 2A). In contrast, the phalloidin-stained actin filaments were localized in the peripheral region (Figure 2B); therefore, Atg12 and actin filaments were not colocalized. This result was confirmed by merging the images (Figure 2C) as well as by the enlarged images of the regions, as indicated by the yellow arrows (D, E) and white arrows (F, G) in Figure 2C. We noticed that the LC3 fluorescence spots were much larger than the Atg12 fluorescence spots (Figure 2H, I).

### 2.3. SE-ADM Analysis of the Localisation of LC3, Atg12 and Actin in Cultured Cells 

**Figure 3:**
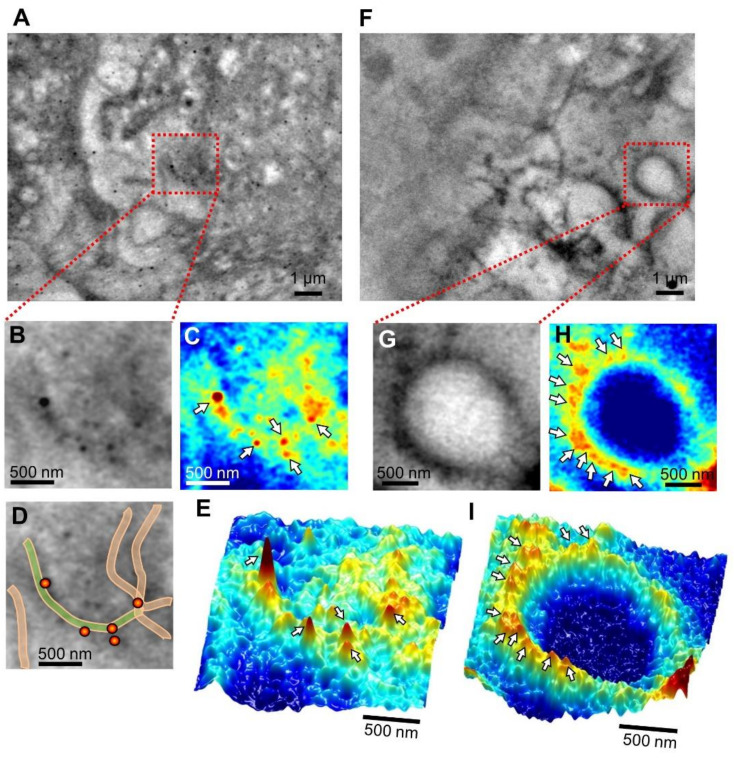
Dielectric images of 4T1E/M3 cells stained with anti-LC3 antibody conjugated to 60 nm colloidal gold particles. (**A**) Dielectric images of the cells stained with anti-LC3 antibody conjugated to 60 nm colloidal gold particles after paraformaldehyde fixation and permeabilization (10,000× magnification, 6 kV electron beam (EB)). (**B**) An enlarged image of the red framed area in (**A**), showing LC3 bound to 60 nm colloidal gold particles. (**C**) A pseudo-color map of (**B**) after intensity inversion. (**D**) A schematic diagram of arc-like structures and colloidal gold particles superimposed on a dielectric image of (**B**). (**E**) A 3D color map of (**C**). (**F**) Another scanning electron-assisted dielectric microscopy (SE-ADM) image of 4T1E/M3 cells (10,000× magnification, 6 kV EB). (**G**) An enlarged image of the red framed area in (**E**). (**H**) A pseudo-color map of (**F**) after intensity inversion. LC3 aligns in a circular manner. (**I**) A 3D color map of (**H**). Scale bars: 1 μm in (**A**, **F**), 500 nm in (**B**–**E**,**G**–**I**).

Cells were cultured on a SiN film placed in an aluminum (Al) holder in a culture dish [22]. After 2–3 h of starvation, the Al holder with the cells on the SiN film was detached and sealed by another acrylic plate and then introduced into the SE-ADM system [21,22]. Prior to observation, the cells were fixed, permeabilized, and labeled with anti-LC3 antibodies and 60 nm colloidal gold particles conjugated to the secondary antibodies. Figure 3 shows an SE-ADM image of the labelled cells. LC3 bound with 60 nm colloidal gold particles formed an arcuate line in the cell (Figure 3A). This arc-like structure is clearly seen in the enlarged image (Figure 3B), pseudo-color map (Figure 3C), schematic diagram (Figure 3D), and 3D pseudo-color map (Figure 3E). An image of another cell region shows a circular structure (Figure 3F–I). Many colloidal gold particles are attached to the structure. Although these arcuate and circular structures have been suggested in other studies [3,6], this is a direct demonstration of the structure in aqueous medium using our SE-ADM method. The control SE-AMD images of 4T1E/M3 cells without starvation is shown in Supplementary Figure S2.

**Figure 4:**
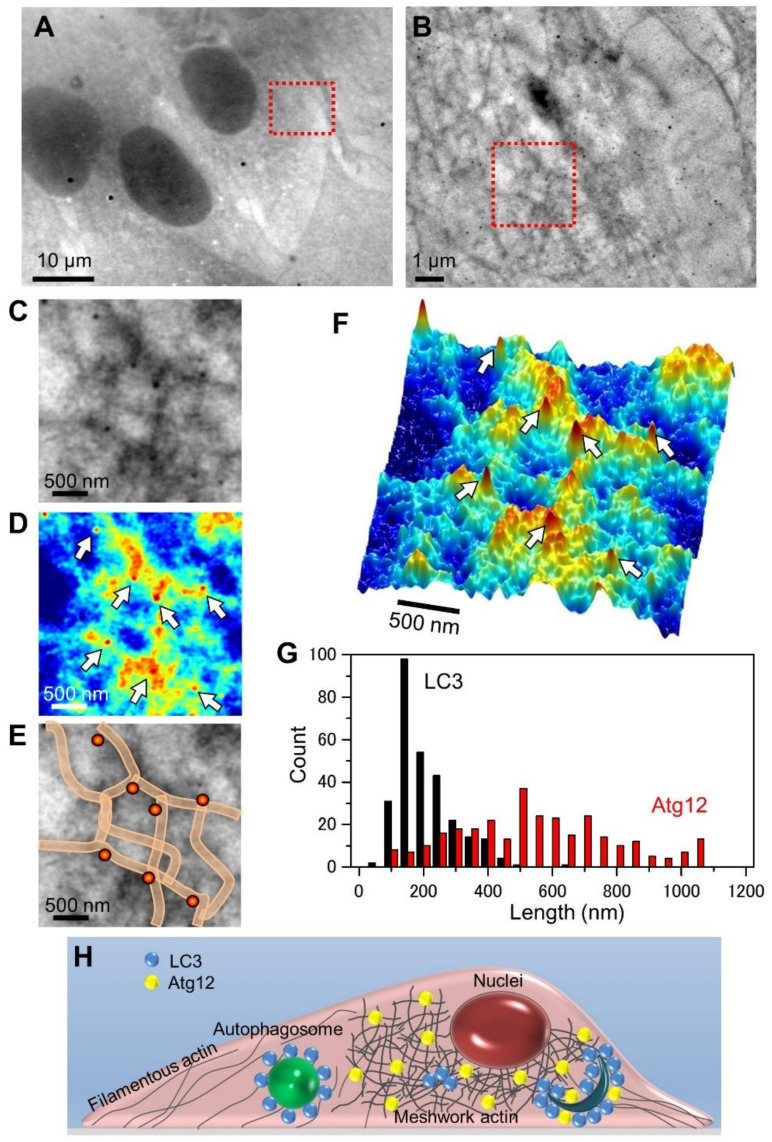
Dielectric images of REF cells stained with anti-Atg12 antibody conjugated to 60 nm colloidal gold particles. (**A**) A low-magnification dielectric image of the cells stained with anti-Atg12 antibody conjugated to 60 nm colloidal gold particles after fixation and permeabilization (1500× magnification, 6 kV EB). (**B**) A high-magnification image (10,000×) of the red framed area in (**A**), showing Atg12 conjugated to 60 nm colloidal gold particles. (**C**) An enlarged image of the red framed area in (**B**). (**D**) A pseudo-color map of (**C**) after intensity inversion. (**E**) A schematic diagram of meshwork structures and colloidal gold particles superimposed on a dielectric image of (**C**). (**F**) A 3D color map of (**D**). (**G**) Histograms of minimum intervals between pairs of colloidal gold particles. Pairs of particles (183 for LS3 and 300 for Atg12) were randomly selected and the intervals were measured. Minimum intervals between LC3-bound colloids are small and sharply peaked, whereas those between Atg12 are larger and gently distributed. (**H**) A schematic illustration of the structural organization of autophagosomes involving LC3 and Atg12 with actin. Scale bars: 10 μm in (**A**), 1 μm in (**B**), 500 nm in (**C**–**F**).

To analyze the localization of Atg12 and the meshwork actin involved in autophagosome formation, we investigated the distribution of Atg12 in the cells using the SE-ADM system (Figure 4). We obtained images of cells labelled with anti-Atg12 antibodies and 60 nm colloidal gold particle-conjugated secondary antibodies. Figure 4A is a low-magnification image (1500×) showing the nucleus, while Figure 4B is a high-magnification image (10,000×) of the area enclosed by the red frame in Figure 4A, focusing on the meshwork structure near the nucleus, because a high expression of Atg12 was detected near the nucleus in fluorescence microscopic images (Figure 2A). In a previous report, we stained cells with anti-actin antibodies and colloidal gold particle-conjugated secondary antibodies. In that report, the meshwork structure was confirmed to be actin [24]. Figure 4C is an enlarged image of the red square in Figure 4B. In Figure 4C–E, Atg12 labelled with 60 nm colloidal gold particles were found to be positioned along the actin meshwork structures. Further, the 3D pseudo-color map clarified the localization of the colloidal gold particles (Figure 4F). Similar results were obtained in other cell regions (Supplementary Figure S3). It was suggested that Atg12 interacted with actin meshwork structures near the nucleus. Our images support the hypothesis that actin plays a direct role in autophagosome formation [3]. 

We show the distribution features of LC3 and Atg12 in the histograms of intervals of two nearest colloidal gold particles from a number of individual measurements (Figure 4G; see also Materials and Methods). The intervals between the gold particles bound to LC3 were mostly less than 400 nm and a sharp peak was observed at 150 nm in the histogram. On the other hand, the intervals between the Atg12-bound colloidal gold particles covered a broad range (100–1100 nm), with a gentle peak at 500 nm (Figure 4G). 

## 3. Discussion

Recent findings suggest that autophagosome formation is mediated and controlled by actin-nucleation promoting proteins [3,18,27]. Actin is essential in a number of cellular processes such as motility, exocytosis and endocytosis, and autophagy [27]. Actin assemblage and autophagy are assumed to take place together, and are thus closely related to each other [3,18,27]. It was demonstrated that actin was necessary for starvation-mediated autophagy and that if actin cytoskeleton was depolymerized, the increase in autophagosome was lost [28].

Our newly developed SE-ADM system produces high-contrast images of autophagy-related components under aqueous conditions with a spatial resolution of 8 nm [21]. This is an obvious advantage because the drying process of specimens in the case of electron microscopy may cause serious structural changes in cell components. The SE-ADM system can examine the cell surface membrane using antibodies against membrane proteins conjugated with gold colloid without fixation. However, at present we have to fix and treat the cells for permeabilization for the introduction of antibodies into the cells to identify the molecules inside the cells.

In this study, we analyzed the relationship between the autophagy-related proteins, LC3, Atg12, and actin, using the SE-AMD system. In a previous study, we clearly observed the actin meshwork structure using our system in mammalian cells under aqueous conditions [24]. The autophagy-related protein Atg12 was clearly seen to be localized on the actin meshwork structure (Figure 4C–E, Supplementary Figure S2) in the SE-ADM images. The Atg12 was broadly dispersed as very small dots in the actin meshwork area in the images of optical microscopy (Figure 2I). On the other hand, LC3 was aligned in an arcuate (Figure 3B–E) or circular (Figure 3G–I) manner near the nucleus. 

The spatial resolution of ordinary fluorescence OM is generally limited to a few hundred nanometres. Therefore, precise and direct observations of the autophagosomes associated with LC3 and Atg12 have been difficult so far (Figure 1 and Figure 2). In contrast, the high-resolution SE-ADM system enabled the observation of the inner cell structures labelled with 60 nm colloidal gold particles and revealed a difference in the localization features between LC3 and Atg12. The intervals between the colloidal gold particles bound to LC3 were about 150 nm, and most of such intervals were less than 400 nm (Figure 4G). In the fluorescence OM images of LC3, this dense distribution caused an appearance of large spots with diameters of around 1 to 3 μm (Figure 2H). In contrast, the colloidal gold particles bound to Atg12 were distributed with longer intervals (100–1100 nm; Figure 4G), so the fluorescent spots were very small (far below 1 μm in diameter) in Figure 2I. Thus, we have been able to demonstrate the distribution characteristics of autophagosome-related proteins, LC3 and Atg12, using the SE-ADM system. 

The SE-ADM system can be used to observe yeast and mammalian cells. Since the system can be easily introduced to regular SEM machines, the wide application of this technique will be possible when it becomes commercially available.

## 4. Materials and Methods

### 4.1. Liquid Samples and the Culture Dish Holder

The liquid sample holder was made as previously described [22]. Briefly, the liquid sample holder was comprised of an upper Al holder and a lower acrylic resin portion that maintains the sample solution at atmospheric pressure between the SiN films [21,22]. A 50 nm-thick SiN film supported by a 0.4 × 0.4 mm window in a Si frame (4 × 4 mm; Silson Ltd., Warwickshire, UK) was coated with tungsten (W) using a magnetron sputtering device (Model MSP-30T, Vacuum Device Inc., Ibaraki, Japan), as previously described [21,22]. The upper W-coated SiN film was attached to the Al holder with pieces of double-sided tape, and the W layer on the SiN film was connected to the Al holder with silver conductive ink. A handmade Al holder with a Si frame was attached beneath a 35 mm culture dish using pieces of double-sided tape to a square hole at the center of the dish, as previously described [22,23]. The culture dish holder was subsequently UV-sterilized for 17–18 h.

### 4.2. Sample Preparation of 4T1E/M3 Cells and Rat Embryonic Fibroblasts

A mouse breast cancer cell line (4T1E/M3) was established as previously described [29,30,31]. The cells were cultured in high-glucose RPMI-1640 medium (Wako, Osaka, Japan) containing 10% fetal bovine serum (FBS, Invitrogen, Thermo Fisher Scientific Inc. Waltham, MA) and 20 mM of HEPES (FUJIFILM Wako Pure Chemical Co. Osaka, Japan) at 37 °C under 5% CO_2_. The medium (1.5 mL/dish) was poured into a culture dish attached to the 50 nm SiN–Al holder. The cells (4 × 104; 20 μL/dish) were seeded and cultured at 37 °C under 5% CO_2_. 

The rat embryonic fibroblasts (REF) cells [26], kindly provided by Keisuke Ohta (Kurume University School of Medicine), were cultured in a low-glucose D-MEM medium (FUJIFILM Wako Pure Chemical Co.) containing 10% fetal bovine serum (FBS), 20 mM of HEPES, and 4 mM of L-glutamine at 37 °C under 5% CO_2_. The cells (4 × 104; 20 μL/dish) were seeded and cultured in 1.5 mL of medium, as described above. The medium was changed after 2–3 days, and the cells formed a sub-confluent or completely confluent monolayer on the SiN membrane in the holder after 3–4 days [22]. Before immunolabelling, the cells were cultured for 2 h with the medium without fetal bovine serum at 37 °C under 5% CO_2_ for starvation to induce autophagy.

### 4.3. Immunofluorescence Staining of the Cells and Observation under an Optical Microscope

All the immunochemical operations were performed at room temperature. The 4T1E/ME3 or REF cells cultured in a 35 mm glass-bottom dish (Matsunami Glass Ind. Ltd., Osaka, Japan) were starved and fixed with PBS containing 4% paraformaldehyde for 10 min, washed twice, and permeabilized in 0.2% Tween 20 for 30 min.

For the double-fluorescence staining of LC3 and actin, the fixed and permeabilized cells in the glass-bottom dishes were stained with mouse anti-LC3 antibody (Medical & Biological Laboratories Co. Ltd. Cat #: M152-3, Clone 4E12, 1/50), diluted in PBS for 30 min, washed twice, and stained with FITC-conjugated anti-mouse IgG antibody (Cat #: 55522, MP Biomedicals Inc., Aurora, OH, USA, 1/100) for 30 min. The cells were then stained with rabbit anti-mouse F-actin antibody (Bioss Inc., Woburn, MA, USA, Cat #: bs-1571R, 1/100) for 30 min, washed twice, and stained with anti-rabbit IgG-conjugated rhodamine (Jackson ImmunoResearch Laboratories Inc. West Grove, PA, USA, Cat #: 711-065-152, 1/100) for 30 min. For phalloidin staining, the cells were stained with the Alexa Fluor 568 phalloidin (Thermo Fisher Scientific Inc. Waltham, MA, USA, A12380, 1/300) for 30 min instead of anti-mouse F-actin and the second antibody. After washing twice, the dish was observed under fluorescence OM (AXIO Observer A1; Carl Zeiss, Oberkochen, Germany). The fluorescence images of the cells were acquired through fluorescence filters at excitation/emission wavelengths of 480/520 nm (green fluorescence of FITC) or 565/620 nm (red florescence of Alexa Fluor or rhodamine).

For the double fluorescence staining of Atg12 and actin, the fixed and permeabilized cells in the glass-bottom dishes were stained with rabbit anti-Atg12 antibody (GNT, Cat#:GTX124181, 1/50) diluted with PBS for 30 min, washed twice, and stained with FITC-conjugated anti-rabbit IgG (Cat #: 55662, MP Biomedicals Inc. Santa Ana, CA, 1/100) for 30 min. The cells were then stained with Alexa Fluor 568 phalloidin (Thermo Fisher Scientific Inc. Waltham, MA, USA, A12380, 1/300) for 30 min. After washing twice, the dish was observed under a fluorescence OM. Fluorescence images of the cells were acquired under the filtering conditions described above for doubly stained LC3 and actin.

### 4.4. Immunolabelling of Cells Using Antibody and 60 nm Colloidal Gold Particles

The cells seeded in the dish holder on the 50 nm SiN film were starved, fixed, and permeabilized in the same way as described above. The cells were stained with mouse anti-LC3 antibody (MBL, Cat#: M152-3, 1/50) for 30 min, washed twice with PBS twice, and stained with anti-mouse IgG-conjugated 60 nm gold colloids (Cytodiagnostics Inc., Burlington, ON, Canada, Cat #: AC-60-02-05, 1/50) for 30 min. After washing twice, the holder was attached to the acrylic sample holder on the 50 nm SiN film and observed with the SE-ADM system [22,23].

For the 60 nm gold labelling of Atg12, the cells were fixed, permeabilized, stained with rabbit anti-Atg12 antibody (GNT, Cat# GTX124181, 1/50) for 30 min, washed twice with PBS, and stained with anti-rabbit IgG-conjugated 60 nm gold colloids (Cytodiagnostics Inc., Burlington, ON, Canada, Cat#: AC-60-17, 1/50) for 30 min.

### 4.5. High-Resolution SE-ADM System and FE-SEM Setup

The high-resolution SE-ADM imaging system was based on field-emission scanning electron microscopy (FE-SEM) (JSM-7000F, JEOL, Tokyo, Japan) and is depicted in Supplementary Figure S1. The liquid sample holder was mounted onto the SEM stage, and the detector terminal was connected to a pre-amplifier under the holder [21]. The electrical signal from the pre-amplifier was passed through a low-pass filter (LPF) and fed into an analog-digital (AD) converter (AIO-163202FX-USB, Contec Co. Ltd., Osaka, Japan), as previously described [21]. The low-pass-filtered and electron beam-scanned signals were logged in a PC through an AD converter at a sampling frequency of 50 kHz. SEM images (1280 × 1020 pixels) were captured under 1000–20,000× magnification with a scanning time of 80 s, a working distance of 7 mm, an EB acceleration voltage of 4–8 kV, and a current of 10 pA.

### 4.6. Image Processing

The SE-ADM signal data from the AD converter were transferred to a personal computer (Intel Core i7, 3.2 GHz, Windows 10). The LPF and scanned signals were processed into high-resolution SE-ADM images using the image-processing toolbox of MATLAB R2018a (Math Works Inc., Natick, MA, USA). The original SE-ADM images were filtered through a 2D Gaussian filter (GF) with a kernel size of 11 × 11 pixels and a radius of 1.2σ. The background was removed by subtracting the SE-ADM images from the filtered images using a broad GF (400 × 400 pixels, 200σ).

### 4.7. Calculation of Minimum Interval Between Colloidal Gold Particles

To calculate the minimal intervals between pairs of colloidal gold particles, we randomly selected 283 and 300 gold particles from the SE-ADM images of cells stained with anti-LC3 antibodies (4 images) and anti-Atg12 antibodies (3 images), respectively. The minimal interval between the colloidal gold particles was calculated as the minimum distance between the (x, y) coordinates of the working colloidal gold particle in the image and the (x, y) coordinates of the other colloidal gold particles. The calculation was performed by MATLAB R2018a running on a PC (Intel Core i7, 3.2 GHz, Windows 10).

## 5. Conclusions

We analyzed the localization of two autophagy-related proteins, LC3 and Atg12, in liquid medium using our newly developed SE-ADM system. We found that during autophagosome formation, Atg12 was localized along the actin meshwork structure, while LC3 formed arcuate or circular alignments. Furthermore, we revealed the different distribution characteristics of LC3 and Atg12. LC3 was distributed with short intervals, while Atg12 was localized in a broad range. The direct observation of cells in a medium using SE-ADM may be useful for further analyses of the formation of autophagosomes.

## Data Availability

The data presented in this study are available on request from the corresponding author.

## Supplementary Materials

The following are available online at https://www.mdpi.com/1422-0067/22/4/1834/s1, Figure S1: Overview of autophagosome observation in cells using the SE-ADM system. (A) A schematic illustration of the SE-ADM system based on FE-SEM with a liquid sample holder. The sample holder containing the cultured cells is mounted on the stage attached to the pre-amplifier. This whole apparatus is introduced to the SEM chamber. (B) A schematic illustration of the liquid holder containing the cells and 60 nm colloidal gold particles bound to LC3 or Atg12. The W-coated SiN film is scanned with the electron beam. The measurement terminal under the holder detects the electrical signal through the cells in the liquid medium. (C) A typical SE-ADM image of the cells in the liquid holder–S3, Figure S2: Dielectric images of 4T1E/M3 cells under normal condition stained with anti-LC3 antibody conjugated to 60 nm colloidal gold particles. (A–D) The high-magnification images of 4T1E/M3 cells stained with 60 nm colloidal gold particles after paraformaldehyde fixation and permeabilisation (10,000× magnification, 10–13 kV EB). Without the treatment of starvation, gold particles are not seen in these SE-ADM images. Scale bars: 1 μm in (A–D), Figure S3: SE-ADM images of REF cells stained with anti-Atg12 antibody conjugated to 60 nm colloidal gold particles. (A) Another low-magnification dielectric image of REF cells stained with anti-Atg12 antibody conjugated to 60 nm colloidal gold particles after paraformaldehyde fixation and permeabilisation (1,000× magnification, 6-kV EB). (B) A high-magnification image (10,000×) of the red framed area in (A), showing Atg12 conjugated to 60 nm colloidal gold particles. (C) An enlarged pseudo-colour map of the red framed area in (B). Atg12 is localised along the meshwork structures. (D) A schematic diagram of meshwork structures and colloidal gold particles superimposed on a dielectric image of (C). (E) A 3D colour map of (D). Scale bars: 10 μm in (A), 1 μm in (B), 500 nm in (C, E).

## Abbreviations

AD	Analog-digital
Al	Aluminum;
Atg12	Conserved autophagy-related gene 12
EB	Electron beam
FITC	Fluorescein isothiocyanate
GF	Gaussian filter
LC3	Microtubule-associated proteins 1A/1B light chain 3
LPF	Low-pass filtered
OM	Optical microscope
REF	Rat embryonic fibroblasts
SE-ADM	Scanning electron-assisted dielectric microscopy
SEM	Scanning electron microscopy
SiN	Silicon nitride
W	Tungsten
