# Supplementary material for: Scanning Electron-Assisted Dielectric Microscopy Reveals Autophagosome Formation by LC3 and ATG12 in Cultured Mammalian Cells
Source: Int J Mol Sci. 2021 Feb 12;22(4):1834. doi: 10.3390/ijms22041834 (PMC7917705; doi:10.3390/ijms22041834)
Supplement: Supplementary file 1 [file ijms-22-01834-s001.pdf]

Supplementary Information

# Scanning electron-assisted dielectric microscopy reveals autophagosome formation by LC3 and ATG12 in cultured mammalian cells

Tomoko Okada, Toshihiko Ogura*

Health and Medical Research Institute, National Institute of Advanced Industrial Science and Technology (AIST), Central 6, Tsukuba, Ibaraki 305-8566, Japan

***Correspondence:** Toshihiko Ogura, Health and Medical Research Institute, National Institute of Advanced Industrial Science and Technology (AIST), Highashi 1-1-1, Tsukuba, Ibaraki 305-8566, Japan

**E-mail**: t-ogura@aist.go.jp

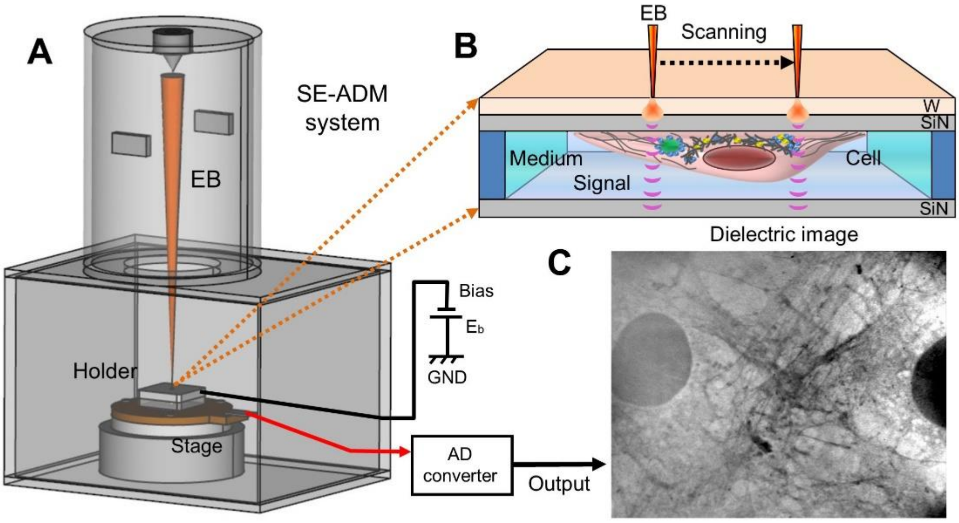


**Supplementary Figure S1**

**Overview of autophagosome observation in cells using the SE-ADM system.** (**A**) A schematic illustration of the SE-ADM system based on FE-SEM with a liquid sample holder. The sample holder containing the cultured cells is mounted on the stage attached to the pre-amplifier. This whole apparatus is introduced to the SEM chamber. (**B**) A schematic illustration of the liquid holder containing the cells and 60 nm colloidal gold particles bound to LC3 or Atg12. The W-coated SiN film is scanned with the electron beam. The measurement terminal under the holder detects the electrical signal through the cells in the liquid medium. (**C**) A typical SE-ADM image of the cells in the liquid holder.

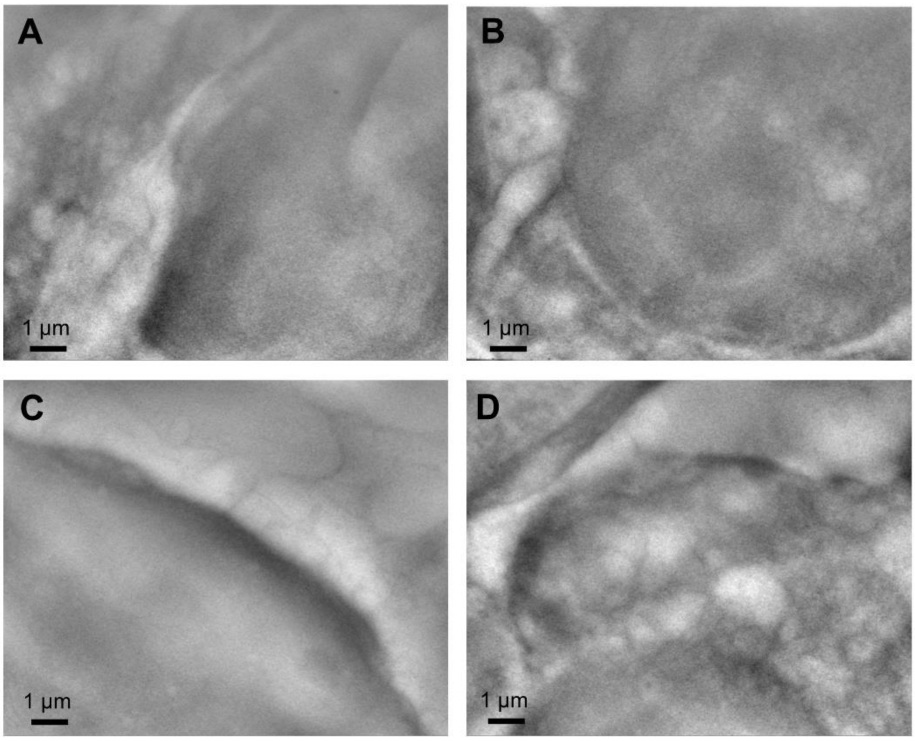


**Supplementary Figure S2**

**Dielectric images of 4T1E/M3 cells under normal condition stained with anti-LC3 antibody conjugated to 60 nm colloidal gold particles.** (**A**–**D**) The high-magnification images of 4T1E/M3 cells stained with 60 nm colloidal gold particles after paraformaldehyde fixation and permeabilisation (10,000× magnification, 10–13 kV EB). Without the treatment of starvation, gold particles are not seen in these SE-ADM images. Scale bars: 1 µm in (A–D).

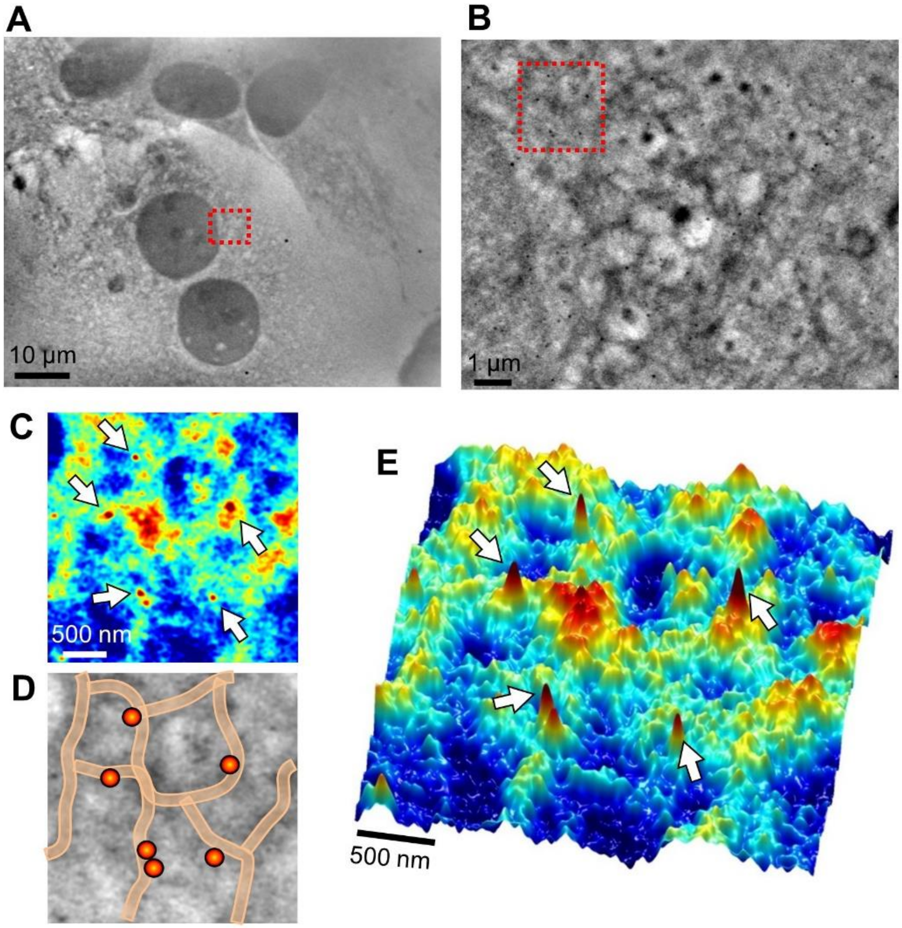


**Supplementary Figure S3**

**SE-ADM images of REF cells stained with anti-Atg12 antibody conjugated to 60 nm colloidal gold particles.** (**A**) Another low-magnification dielectric image of REF cells stained with anti-Atg12 antibody conjugated to 60 nm colloidal gold particles after paraformaldehyde fixation and permeabilisation (1,000× magnification, 6-kV EB). (**B**) A high-magnification image (10,000×) of the red framed area in (A), showing Atg12 conjugated to 60 nm colloidal gold particles. (**C**) An enlarged pseudo-colour map of the red framed area in (B). Atg12 is localised along the meshwork structures. (**D**) A schematic diagram of meshwork structures and colloidal gold particles superimposed on a dielectric image of (C). (**E**) A 3D colour map of (D). Scale bars: 10 µm in (A), 1 µm in (B), 500 nm in (C, E).
